# Supplementary material for: Who gets missed in Zambia’s antenatal HIV testing? Persistent inequities despite routine screening policies in Zambia
Source: PLOS Glob Public Health. 2025 Dec 1;5(12):e0004972. doi: 10.1371/journal.pgph.0004972 (PMC12668532; doi:10.1371/journal.pgph.0004972)
Supplement: S1 Table — (DOCX) [file pgph.0004972.s001.docx]

**S1 Table. Multivariable Logistic Regression with Interaction Terms for HIV Testing During Pregnancy, Zambia DHS 2007–2018**

| **Characteristic** | **aOR** | **95% CI** | **p-value** |
| --- | --- | --- | --- |
| **Survey Year** |  |  |  |
| 2007 (Ref) | — | — | — |
| 2013 | 2.26 | 1.93–2.65 | <0.001 |
| 2018 | 3.00 | 2.50–3.61 | <0.001 |
| **Age Group** |  |  |  |
| <20 (Ref) | — | — | — |
| 20–34 | 1.49 | 1.23–1.80 | <0.001 |
| 35+ | 1.44 | 1.11–1.87 | 0.008 |
| **Education Level** |  |  |  |
| No education (Ref) | — | — | — |
| Primary | 1.70 | 1.39–2.09 | <0.001 |
| Secondary | 2.59 | 2.04–3.28 | <0.001 |
| Higher | 2.31 | 0.77–6.94 | 0.13 |
| **Residence** |  |  |  |
| Rural (Ref) | — | — | — |
| Urban | 1.51 | 0.61–3.74 | 0.37 |
| **Wealth Quintile** |  |  |  |
| Poorest (Ref) | — | — | — |
| Poorer | 1.38 | 1.14–1.67 | <0.001 |
| Middle | 1.71 | 1.39–2.11 | <0.001 |
| Richer | 1.93 | 1.42–2.64 | <0.001 |
| Richest | 1.41 | 0.64–3.11 | 0.40 |
| **Interactions** |  |  |  |
| Primary × Urban | 0.54 | 0.28–1.04 | 0.066 |
| Secondary × Urban | 0.45 | 0.23–0.88 | 0.017 |
| Higher × Urban | 0.41 | 0.11–1.54 | 0.19 |
| Poorer × Urban | 1.78 | 0.80–3.95 | 0.17 |
| Middle × Urban | 2.13 | 0.97–4.67 | 0.060 |
| Richer × Urban | 0.99 | 0.38–2.54 | 0.99 |
| Richest × Urban | 1.39 | 0.47–4.15 | 0.55 |

*Notes:*
aOR = adjusted odds ratio; CI = confidence interval; Ref = reference category. Models are adjusted for survey design (weights, clusters, and strata). Interaction terms assess whether education and wealth effects differ by urban versus rural residence.
