# Supplementary material for: Who gets missed in Zambia’s antenatal HIV testing? Persistent inequities despite routine screening policies in Zambia
Source: PLOS Glob Public Health. 2025 Dec 1;5(12):e0004972. doi: 10.1371/journal.pgph.0004972 (PMC12668532; doi:10.1371/journal.pgph.0004972)
Supplement: Attachment — Submitted filename: Rebuttal Letter_Who gets missed manuscript.docx [file pgph.0004972.s003.docx]

**Dear Dr. Jimah and Reviewers,**

We would like to sincerely thank you for your thoughtful and constructive feedback on our manuscript entitled *“Who Gets Missed? Inequities in Antenatal HIV Testing in Zambia Despite Routine Screening.”* We are grateful for the opportunity to revise and resubmit to PLOS Global Public Health. The detailed comments have been invaluable in strengthening our analysis, presentation, and interpretation. Below, we provide a point-by-point response, with each reviewer’s comment quoted in italics, followed by our response. Line numbers refer to the revised tracked manuscript.

**Editorial Comments**

**Comment 1:** Please amend your detailed Financial Disclosure statement. This is published with the article. It must therefore be completed in full sentences and contain the exact wording you wish to be published.

**Response:** We have revised the Financial Disclosure statement as requested. The final version now reads: *“***The authors received no specific funding for this work.***”* (Line 354).

**Comment 2:** Please provide separate figure files in .tif or .eps format.

**Response:** We have prepared and uploaded all figures as separate high-resolution .tif files in accordance with PLOS guidelines.

**Comment 3:** If the reviewer comments include a recommendation to cite specific previously published works, please review and evaluate these publications to determine whether they are relevant and should be cited.

**Response:** We reviewed all references and incorporated those that were directly relevant.

**Comment 4:** Please review your reference list to ensure it is complete and correct.

**Response:** We carefully checked all references and ensured accuracy in formatting and completeness. We also confirmed that none of the cited works were retracted.

**Reviewer #1**

**Abstract**

- ***“*Consider adding the sample size and clarifying that HIV testing was self-reported.”**
  **Response:** We have added the sample size (N = 4047 in 2007, 9,190 in 2013/2014, and 7245 in 2018) and clarified that HIV testing was self-reported (Lines 50–51).
- “The conclusion is strong. If possible, include a brief mention of specific strategies.”
  **Response:** We now highlight mobile clinics and peer-led education as strategies shown to work in other sub-Saharan African settings in the conclusion of the abstract (Lines 62–63).

**Introduction**

- **“When introducing the topic of inequities, briefly name mechanisms such as stigma, male partner influence, structural barriers.”**
  **Response:** We expanded the introduction to specify key mechanisms underlying inequities in HIV testing, including stigma, male partner influence, and structural barriers (Lines 87–89).

**Methods**

- **“Please clarify whether the HIV testing outcome includes both receipt of the test and receipt of the result.”**
  **Response:** We clarified that the outcome measure was based on self-reported receipt of an HIV test during pregnancy, not necessarily receipt of results (Lines 127-128).
- **“Briefly explain the quasi-binomial error structure.”**
  **Response:** We added a short explanation for using a quasi-binomial model to account for overdispersion (Lines 144-145).
- **“Consider reporting diagnostics or model performance measures.”**
  **Response:** We now report McFadden’s pseudo-R² and area under the ROC curve (AUC = 0.66), along with variance inflation factor (VIF) checks for multicollinearity (Lines 146–148).

**Results**

- **“Consider adding a row summarizing HIV testing rates per year in Table 1.”**
  **Response:** We revised Table 1 to include HIV testing rates by survey year.
- **“One extension may be testing interaction terms between predictors and place of residence.”**
  **Response:** We conducted additional interaction analyses. Results are reported in the main text (235-240) and illustrated in supplementary Table S1.
- **“A forest plot may improve visual accessibility.”**
  **Response:** We added a forest plot of adjusted odds ratios (Figure 2) for improved clarity (Lines 208–213).

**Discussion**

- **“Suggest school-based HIV education or adolescent ANC peer navigators.”**
  **Response:** We added recommendations for adolescent-focused strategies, including peer navigators and school-based outreach (Lines 278–284).
- **“Consider adding a comment about ensuring reliable supply chains in rural clinics.”
  Response:** We emphasized the importance of reliable supply chains and health system trust in the discussion (Lines 299–302).
- **“Emphasize that even among women attending ANC, disparities persist.”
  Response:** We highlighted this as a key policy implication, underscoring the need for “last-mile” interventions targeting already-engaged populations (Lines 263–274, 320–321).

**Limitations and Strengths**

- **“Note that social desirability bias may vary by setting.”**
  **Response:** We revised the limitations to specify that social desirability bias may differ by context and could lead to underestimation of disparities (Lines 308–309).
- **“Rephrase repetitive expressions (‘less educated, poorer, and rural-dwelling’).”
  Response:** We rephrased for conciseness, now using “lower socioeconomic status and residing in rural areas” (Lines 325–326).
- **“Use consistent terminology for ANC after first introduction.”**
  **Response:** We corrected abbreviation use across the manuscript to consistently use “ANC.”

**Reviewer #2**

- **Comment:** **“Very nice use of DHS surveys… persistent gaps remain… some redundancy between tables and figures, but still helpful.”**
- **Response:** We thank the reviewer for their supportive assessment. We carefully reviewed tables and figures to avoid redundancy while retaining those that add clarity. Forest plots and predicted probability graphs were added to strengthen accessibility (Figures 2 & 4).

We are grateful to the editor and reviewers for their constructive feedback. We believe the revisions have substantially strengthened the manuscript, improving methodological transparency, policy relevance, and readability. We hope the revised version now meets the standards of PLOS Global Public Health*.*

Sincerely,
Newton Nyirenda, MD, MSc (Epidemiology)
On behalf of all co-authors
